# The involvement of the dysfunctional insulin receptor signaling system in long COVID patients with diabetes and chronic pain and its implications for the clinical management using taVNS

**DOI:** 10.3389/fpain.2024.1486851

**Published:** 2024-11-25

**Authors:** Riwang Li, Wenguo Liu, Dahai Liu, Xu Jin, Shuxing Wang

**Affiliations:** ^1^ Department of Anatomy, Medical School, Foshan University, Foshan, China; ^2^ Department of Anesthesiology, Cancer Hospital Chinese Academy of Medical Sciences, Beijing, China

**Keywords:** long COVID, sequelae, chronic pain, type 2 diabetes mellitus, insulin receptor, taVNS, vagal nerve stimulation

## Abstract

In clinical terms, chronic pain is the most prevalent sequela resulting from COVID-19, which is induced by the novel coronavirus (SARS-CoV-2), while type 2 diabetes mellitus (T2D) is the most common comorbidity. This triangular relationship can be attributed to the dysfunction of the insulin receptor signaling system (IRSS) in both central and peripheral systems. Patients with T2D are essentially more susceptible to SARS-CoV-2 infection due to the widespread expression of angiotensin converting enzyme 2 (ACE2) in their pancreatic beta cells, which serves as the cellular port for the SARS-CoV-2 to infect and enter the cell. This infection can exacerbate chronic pain and insulin resistance for various reasons. Peripherally, once infected, the virus can cause damage to peripheral nerves and pancreatic β-cells, further exacerbating pain and glucose metabolism conditions. Additionally, in the central nervous system, dysfunctional IRSS is closely linked to chronic pain. Over the past few years of the COVID-19 pandemic, an increasing body of evidence suggests that insulin and other medications currently used in clinical practice for hyperglycemia control may not be safe for treating these patients. Therefore, we need a proper approach for the treatment of chronic pain in long COVID patients, especially patients with T2D. This review presents evidence that transcutaneous auricular vagal nerve stimulation (taVNS) may provide a viable treatment option for chronic pain and metabolic dysfunction by improving the function of IRSS in both the central nervous system and peripheral tissues.

## Introduction

Pain is an unpleasant sensory and emotional experience associated with, or resembling that associated with, actual or potential tissue damage (1). It is typically categorized into acute and chronic pain. Acute pain serves as a physiological adaptation and protection mechanism, alerting the body to critical tissue damage. Typically, once the initial destructive or nociceptive stimulus is removed and the injury has healed, the pain will subside. In some cases, despite the apparent absence of a local stimulus, the pain persists for years or even a lifetime (2). This type of persistent pain, lacking an identifiable source of stimulation and persisting beyond the anticipated recovery period, is designated as chronic pain (1). At present, chronic pain is recognized as a distinct disease entity, characterized by four primary symptoms: spontaneous pain, hyperalgesia (increased sensitivity to pain stimuli), allodynia (pain triggered by normally harmless stimuli), and the abnormal perception of pain in unaffected areas of the body.

Since its outbreak at the end of 2019, Coronavirus disease 2019 (COVID-19), caused by the novel coronavirus SARS-CoV-2, has caused substantial morbidity and mortality worldwide. A significant proportion of patients who have recovered from SARS-CoV-2-induced viral illness often report a range of clinical symptoms, despite biochemical evidence indicating the cessation of SARS-CoV-2 replication 4 or more weeks post-initial infection. These post-acute sequelae of SARS-CoV-2, also known as long COVID, are defined as signs, symptoms, and conditions that persist or emerge following an initial COVID-19 infection, and can persist for weeks, months, or even years. Moreover, the post-acute sequelae of SARS-CoV-2-related symptoms are diverse and can manifest in physical, psychological, and cognitive aspects, such as sensory dysfunction and cognitive-communication difficulties. Out of all the sequelae resulting from SARS-CoV-2 infection, chronic pain is the most prevalent (3). According to the report, 18% of COVID-19 survivors who had been previously hospitalized experienced multitype pain as a new symptom after recovering from COVID-19, more than a year following their hospital discharge (4). Chronic pain commonly affects up to 77% of infected individuals and may serve as a significant factor in impacting their ability to return to work and the quality of their life within 5 years following discharge (3). Therefore, exploring the mechanism and discovering effective approaches for preventing and treating chronic pain in patients with long COVID is of great significance. In this review, we present evidence that taVNS may serve as a viable treatment option for chronic pain and metabolic dysfunction in patients with long-term COVID-19 and type 2 diabetes.

## Patients infected with SARS-CoV-2 are predisposed to experiencing chronic pain

Patients infected with the SARS-CoV-2 are highly susceptible to chronic pain due to several factors. (1) Acute pain is a common risk factor for chronic pain. SARS-CoV-2 infection often causes pain, such as myalgia, arthralgia, abdominal pain, headache, and chest pain. Additionally, critically ill patients in the ICU may undergo procedures like tracheotomy and intubation, which can be extremely painful. Based on the condition, some patients may undergo multiple pain-related interventions; however, the more pain they experience, the higher the risk of developing chronic pain after discharge (5). (2) Survivors of SARS-CoV-2 infection frequently endure prolonged periods of fixation, sedation, and mechanical ventilation (6), which necessitate the administration of neuromuscular blockers (7). Regrettably, the targets of these medications encompass not only motor functions but also sensory components, potentially precipitating conditions that can induce chronic pain, including polyneuropathy, critical myopathy, muscle atrophy, joint pain, and contractures (8). (3) One method of respiratory support for SARS-CoV-2 infected patients is to repeatedly place the patient in the prone position to enhance ventilation (9). However, complications of the prone position under sedation include brachial plexus disease, joint subluxation, and soft tissue injury, all of which can result in chronic neuralgia and musculoskeletal pain (10). (4) Both the SARS-CoV of 2003 and the herpes zoster virus have the ability to invade the nervous system, potentially leading to polyneuropathy (11). It is possible that through a similar mechanism, the novel coronavirus, specifically the SARS-CoV-2 of 2019, can also invade the nervous system and induce symptoms such as confusion, headache, dizziness, and a loss of taste or smell (12). Additionally, it has been observed to induce abdominal pain similar to that experienced in Guillain-Barre syndrome (13), as well as neuropathic symptoms resembling Miller Fisher syndrome (14). (5) Thrombosis, hypotension, and hypoxemia, which are caused by SARS-CoV-2 infection, can trigger neuralgia (3). (6) Emotional conditions such as depression, anxiety, distress, and insomnia, resulting from quarantine or isolation, along with Post-Traumatic Stress Disorder (PTSD), are all known to be associated with chronic pain (15).

## SARS-CoV-2 infection is linked to a hypofunctioning insulin receptor signaling system

Evidence suggests that there is a bidirectional relationship between SARS-CoV-2 disease and T2D, as these two conditions mutually exacerbate each other (16). Diabetes mellitus, primarily in the form of T2D, is one of the most prevalent comorbidities associated with SARS-CoV-2 infection, affecting between 7% and 33% of both confirmed cases and asymptomatic individuals. Notably, patients with T2D are at an elevated risk of developing severe complications, experiencing more pronounced pneumonia, and facing higher hospitalization and mortality rates when compared to their non-diabetic counterparts infected with the SARS-CoV-2 (16).

**Figure 1:**
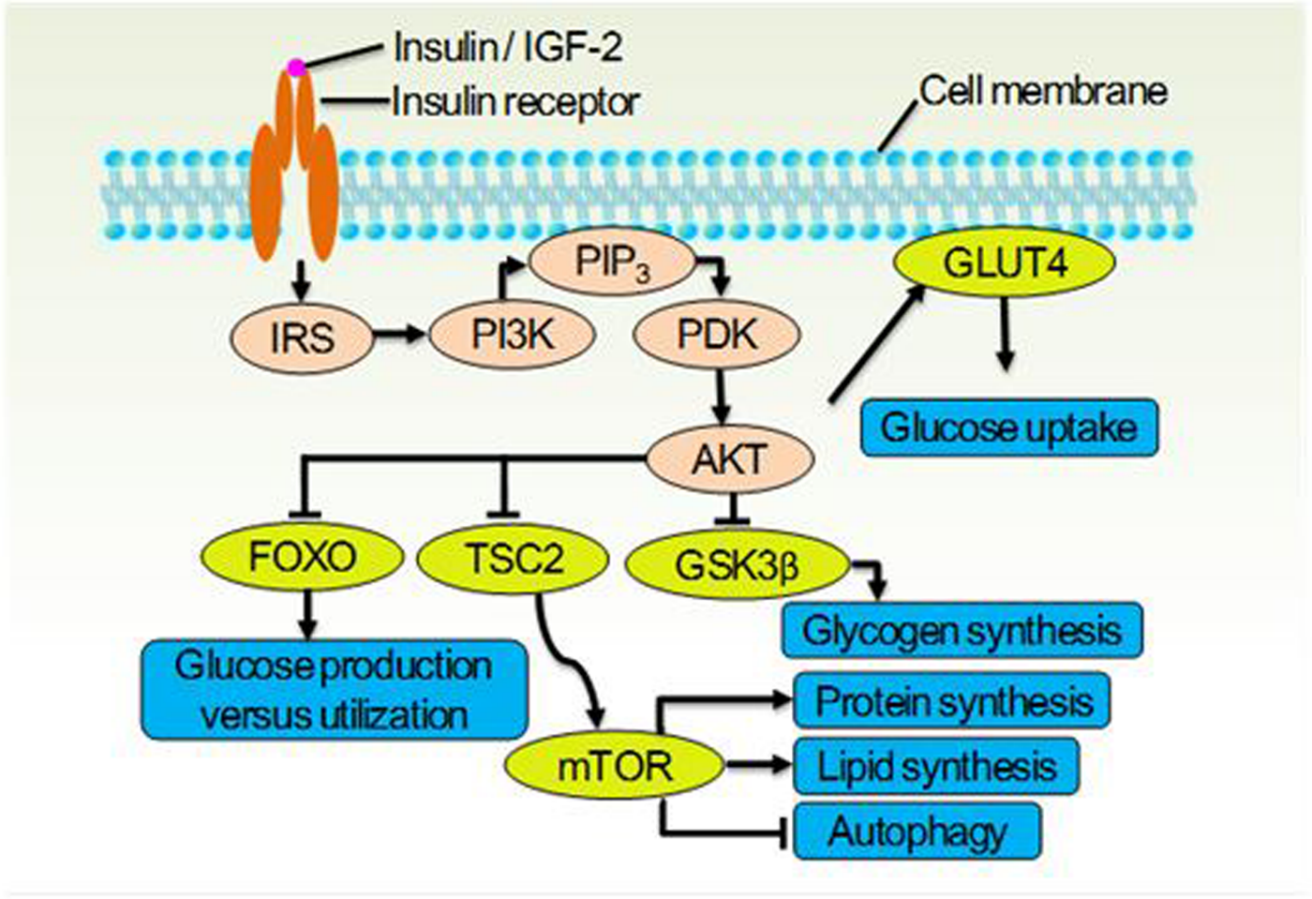
The insulin receptor signaling system (IRSS) and partial of its physiological function. Once the insulin receptor (IR) tyrosine kinase is activated by insulin or IGF-2, it causes tyrosine phosphorylation of IR and of the IR substrate (IRS) proteins. Phosphotyrosine sites on IRS allow binding of the lipid kinase PI3K, which synthesizes PtdIns(3,4,5)P_3_ (PIP_3_) at the cytomembrane. This recruits the phosphoinositide-dependent kinase (PDK), which directly phosphorylates the Thr308 residue of AKT. Activated AKT goes on to translocate GLUT4 to cytomembrane thus let glucose entering the cell, and inhibit the glycogen synthase kinase 3β (GSK3β), the forkhead family box O (FOXO) transcription factors, and the protein tuberous sclerosis 2 (TSC2) which permits activation of mTOR. These effector proteins mediate the effects of insulin on glucose production, utilization and uptake, the synthesis of glycogen, protein and lipid, as well as the cellular autophagy.

**Figure 2:**
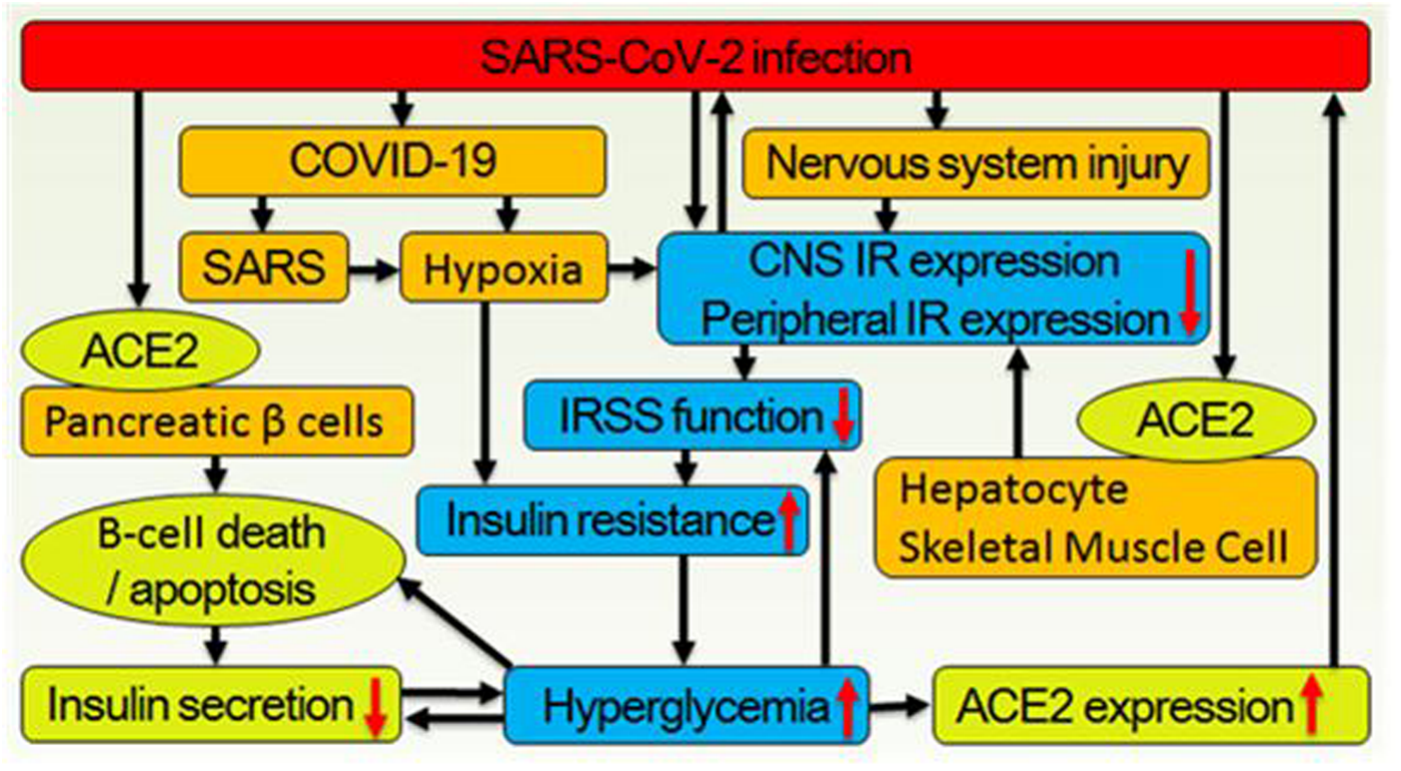
Correlations between the SARS-CoV-2 infection and hyperglycemia. (1) Virus binds to ACE2 on the cytomembrane surface of pancreatic β-cell and enters it thus leading to β-cell death or apoptosis, which decrease insulin secretion. Meanwhile, virus infecting neural, hepatic, and skeletal muscle cells lead to downregulation of central and peripheral insulin receptor expression. (2) As the main symptom of COVID-19, hypoxia also leads to systemic insulin resistance. (3) Decreased secretion of insulin and downregulated expression of systemic insulin receptor deteriorate glucose metabolism further. (4) Hyperglycemia simultaneously inhibits IRSS function and reduces insulin expression, biosynthesis, secretion, and increases β cell apoptosis. (5) Hyperglycemia also increases ACE2 expression thus increases SARS-CoV-2 infection/reinfection risk.

The insulin receptor (IR), which belongs to the receptor tyrosine kinase family, undergoes conformational changes upon activation. This activates the insulin receptor signaling system (IRSS) (Figure 1) and subsequently regulates various cellular processes such as metabolism, survival, protein synthesis, and other activities (17). The dysfunctional IRSS is a crucial factor in the pathology associated with T2D and insulin resistance (InsR), and it also serves as the primary cause for the mutually reinforcing relationship between SARS-CoV-2 infection and T2D, as depicted in Figure 2. On the one hand, angiotensin converting enzyme 2 (ACE2), the primary cellular binding target and entry port for SARS-CoV-2 to infect the body, is extensively expressed in human pancreatic β-cells. As a result, the virus can directly infect these cells, leading to their death (18) or inducing apoptosis (19). Either way, a reduced insulin secretion and circulating level will ensue. (1) SARS-CoV-2 infection negatively impacts insulin sensitivity and β-cell function (16). (2) Novel SARS-CoV-2 can aggravate InsR by attacking a variety of metabolic organs including the liver and skeletal muscle (20). (3) Hypoxia downregulates IR expression and exacerbates InsR (21), while the main clinical feature of SARS-CoV-2 infection is exactly hypoxia. In individuals with T2D, these results would further aggravate the preexisting glucometabolic perturbations, while in non-diabetic and InsR individuals, it might induce T2D (21). On the other hand, (1) hyperglycemia leads to elevated expression of ACE2 in lungs and other tissues (22). (2) In immune cells, hypofunction of IRSS can directly lead to defective immune responses and SARS-CoV-2 susceptibility (23). (3) Patients with T2D have increased preexisting and potential inflammatory levels associated with InsR, which will enhance inflammatory responses upon SARS-CoV-2 infection, causing extreme systemic immune response “cytokine storm” and onset of acute respiratory distress syndrome (23, 24).

## Nerve injury exacerbates glucose metabolism and neuralgia by inhibiting IRSS function

Physiologically, an effective activation of IRSS depends on the cascading reactions of ligands (insulin or IGF-2), IR, and downstream molecules. Insufficient ligands or downregulated IR expression can lead to a loss of full control over downstream factors such as PI3K, AKT, mTOR, and GSK-3b, ultimately promoting chronic pain (25) (Figure 1). It is an undisputed fact that IRSS hypofunction leads to painful diabetic neuropathy (25). Among the multiple factors that negatively affect the IRSS function in diabetic individuals, both hyperglycemia (26–28) and downregulated expression of IR caused by hyperglycemia are well verified (29–31).

A hypofunction of IRSS not only deteriorates glucose metabolism but also exacerbates chronic pain. A preclinical comparative study on little mates of Zucker diabetic fatty (ZDF) and Zucker lean rats (ZL) revealed that, although there appeared to be no difference at birth, adult ZDF rats had a lower baseline pain threshold accompanied by a reduced IRSS function (32). Moreover, in ZDF pain model rats produced by constrictive sciatic nerve injury (CCI), the progression of diabetes was significantly accelerated, as evidenced by elevated fasting blood glucose levels, plasma HbA1c, and insulin. Notably, the pain behavior following CCI closely correlated with the progression of diabetes, with a significant negative correlation observed between blood glucose concentration and pain threshold (32). Meanwhile, 2 weeks after the operation, the number of IR immunopositive neurons in the posterior horn of the spinal cord ipsilateral to CCI significantly decreased. Additionally, the level of IR protein in the skeletal muscle tissue innervated by the injured nerve was also significantly lower than on the contralateral side (32). In individuals with diabetes, SARS-CoV-2 -infected tissue may be widespread, affecting the skeletal muscle of the entire body as well as the liver. The disturbances in InsR and glucose metabolism may be more severe than in rats with CCI. According to the results obtained from the ZDF and CCI model, the deterioration of IRSS function in diabetic individuals is primarily attributed to InsR rather than insulin secretion. This finding aligns with the research conducted by other scholars, who have demonstrated that InsR, rather than cell damage, plays a significant role in the rapid metabolic deterioration of T2D or the emergence of new hyperglycemia following SARS-CoV-2 infection (20). It is possible that the deterioration of neuralgia and diabetes induced by CCI also exists in nerve injuries following the invasion of herpes zoster virus and coronavirus including SARS-CoV-2, encompassing both mechanism and phenomenon aspects (11, 33).

## Central IRSS hypofunction is linked to chronic pain

In the central nervous system (CNS), the IRSS participate in the mRNA translation, thereby influencing synapse formation and neuroplasticity (34). The neuroplasticity, triggered by aberrant protein translation, is intricately linked to the mechanisms and ramifications of chronic pain. This connection involves modifications in molecular structure and attributes, altered ion channel and electrophysiological properties, neuronal atrophy, synaptic connectivity, and circuit reorganization. Consequently, this leads to modified brain structure and functionality, as well as modified pain behavior resulting from these modifications (35–37). On the contrary, effective pain treatment or the inhibition of abnormal protein translation has the potential to reverse this association (38).

In addition to these pain-related changes, prolonged IRSS hypofunction also impacts intracellular glucose homeostasis, resulting in ATP deficiency or depletion. Consequently, the affected neurons may exhibit hyperexcitability due to insufficient energy to sustain the transmembrane K+/Na+ concentration gradient and a normal resting membrane potential (39, 40). Furthermore, an energy deficiency can cause a reduction in dendritic spines and synaptic plasticity (41), disruption in synaptic transmission (42, 43), and ultimately result in the formation of synaptic “pain memory” (44), leading to a sensation of “continuous pain” in the cortex (45).

## Transcutaneous auricular vagal nerve stimulation (taVNS) enhances both central and peripheral IRSS function

Recently, numerous studies have established a link between chronic pain and dysfunction of the autonomic nervous system, leading to the emergence of various methods for pain relief through parasympathetic excitation. Our research has identified taVNS as a promising approach with verified curative effects. In tests on rats with nerve injuries, taVNS demonstrated both immediate and long-term beneficial effects. Immediately, upon initiation of each taVNS session, a rhythmic and fluctuating secretion of melatonin and insulin was promptly induced, commencing with the onset of stimulation and persisting for a minimum of 2 h following the cessation of stimulation (46). Physiologically, this is the optimal secretion mode for both hormones to exert their full efficacy. Regarding the long-term effects, rats with a pain model showed significant upregulation of central (amygdala, spinal dorsal horn) and peripheral (liver, skeletal muscle) IR expression, alleviation of pain behavior, and improvement in glucose metabolism after receiving taVNS once daily for a consecutive 4-week period.

## Conclusion

At present, it is evident that: (1) Hypofunctional IRSS is implicated in InsR, T2D, chronic pain, and SARS-CoV-2 infection; (2) certain antidiabetic medications may upregulate ACE2, thereby elevating the risk of SARS-CoV-2 infection (16, 20, 21); (3) insulin, as the primary choice (47) for managing acute hyperglycemia in patients infected with SARS-CoV-2, its dosage may contribute to a less favorable prognosis of the SARS-CoV-2 infection (22, 48), (4) The binding between insulin and its receptor is classified as negative cooperative binding (49), which exhibits allosteric inhibition characteristics. Due to this binding nature, when an insulin molecule binds to an IR molecule, the receptor undergoes allosteric changes that decrease its affinity, thereby reducing the likelihood of other insulin molecules binding to the same receptor (50). Furthermore, introducing new insulin into the microenvironment at this juncture will expedite the release of the pre-existing insulin from its receptor (50). (5) Given that the expression of IR is inversely modulated by insulin, continuous insulin supplementation will result in a further suppression of IR expression (51). (6) The taVNS can upregulate central and peripheral IR expression, relief chronic pain, and improve glucose metabolism. And (7) Clinically and pre-clinically, taVNS has been proven to be safe and effective in treating pain, glucose metabolic disorders, and conditions exacerbating chronic pain, such as depression, insomnia, stroke, anxiety, and fear (52). In light of these facts, taVNS may be deemed as an appropriate approach for managing chronic pain in patients who are insulin resistant, particularly for the prevention and treatment of chronic pain following SARS-CoV-2 infection.

## Data Availability

The original contributions presented in the study are included in the article/Supplementary Material, further inquiries can be directed to the corresponding authors.
